# Minimal approach to neuro-inspired information processing

**DOI:** 10.3389/fncom.2015.00068

**Published:** 2015-06-02

**Authors:** Miguel C. Soriano, Daniel Brunner, Miguel Escalona-Morán, Claudio R. Mirasso, Ingo Fischer

**Affiliations:** Instituto de Física Interdisciplinar y Sistemas Complejos, (UIB-CSIC)Palma de Mallorca, Spain

**Keywords:** information processing, dynamical systems, delay, reservoir computing, pattern recognition, machine-learning, hardware, photonics

## Abstract

To learn and mimic how the brain processes information has been a major research challenge for decades. Despite the efforts, little is known on how we encode, maintain and retrieve information. One of the hypothesis assumes that transient states are generated in our intricate network of neurons when the brain is stimulated by a sensory input. Based on this idea, powerful computational schemes have been developed. These schemes, known as machine-learning techniques, include artificial neural networks, support vector machine and reservoir computing, among others. In this paper, we concentrate on the reservoir computing (RC) technique using delay-coupled systems. Unlike traditional RC, where the information is processed in large recurrent networks of interconnected artificial neurons, we choose a minimal design, implemented via a simple nonlinear dynamical system subject to a self-feedback loop with delay. This design is not intended to represent an actual brain circuit, but aims at finding the minimum ingredients that allow developing an efficient information processor. This simple scheme not only allows us to address fundamental questions but also permits simple hardware implementations. By reducing the neuro-inspired reservoir computing approach to its bare essentials, we find that nonlinear transient responses of the simple dynamical system enable the processing of information with excellent performance and at unprecedented speed. We specifically explore different hardware implementations and, by that, we learn about the role of nonlinearity, noise, system responses, connectivity structure, and the quality of projection onto the required high-dimensional state space. Besides the relevance for the understanding of basic mechanisms, this scheme opens direct technological opportunities that could not be addressed with previous approaches.

## 1. Introduction

### 1.1. Introduction to reservoir computing

Recurrent neural networks (RNNs), characterized by the existence of closed loops, are ubiquitous in the brain. Therefore, RNNs are being employed for a family of machine-learning approaches that have been inspired by the way our brain seems to process information. The neuro-inspired approach of RNNs relies on the property that these systems are able to learn from examples, and to process information within a temporal and spatial context (Buonomano and Maass, 2009). RNNs have been proven very powerful to solve certain problems that are computationally hard for standard computers. However, the optimization or training procedure typically involves the calibration of numerous parameters, including the connections between network nodes and their weights. This procedure, that typically depends on the task, is very time-consuming.

In an early work of Buonomano and Merzenich (1995), a new framework for neuronal computation was proposed to perform temporal and spatiotemporal information processing. This approach uses a hidden random recurrent network, which is left untrained, and that is processed by a simple classification/regression technique. Maass and coworkers proposed a similar framework in 2002 (Maass et al., 2002), with the aim of processing sensory inputs in real time by using recurrent neural circuits. Their approach, known as liquid state machine, offers a solution to reduce the complexity of training RNNs, while keeping the capability to perform context-dependent computations (Verstraeten et al., 2007; Lukoševič et al., 2012). Almost at the same time, a similar approach, known as echo state networks, was independently developed by Jaeger and Haas (2004). All these models make use of the transient states generated by high-dimensional dynamical systems combined with a simple learning procedure. The unification of echo state networks and liquid state machine is nowadays known as reservoir computing (Verstraeten et al., 2007).

The implementation of reservoir computing is generally composed of three distinct parts: an input layer, the reservoir and an output layer. Thus, the operating principle of reservoir computing can be summarized as follows. The input sensory information to be processed is connected via an input layer to a RNN, in this context referred to as the reservoir. The projection weights between the sensory input and the neurons in the reservoir are often chosen randomly, although they can also be optimized deterministically (Appeltant et al., 2014). In the reservoir, the connections between neurons (or nodes) are fixed and remain fixed with a certain coupling topology, also often chosen at random. Due to the input signal, the nodes of the reservoir remain in a transient state such that each input is injected in the presence of the response to the previous input. In this way, one benefits from the ability of the system to analyze information with temporal context. The activity of the neurons in the reservoir is read out via an output layer, with connection weights that are trained for the specific task. Adjusting these weights during the training/learning procedure is typically performed by a simple linear regression, i.e., using linear classifiers (Lukoševič et al., 2012). Thus, the training/learning procedure is drastically simplified. The computational performance of the system as a whole relies on the nonlinear mapping of the input signal onto the reservoir state, its sufficiently-high dimensionality, and the ability to properly adjust the readout weights.

The neuro-scientific reality of the reservoir computing has already been envisioned in the work of Maass et al. (2002). The basic principles of RC are indeed based on the way our brain processes, or at least seems to process, information. Moreover, subsequent studies exist that discuss the neurophysiological reality of this scheme further. In 2007, Yamazaki and Tanaka proposed a RC-like model for the cerebellum (Yamazaki and Tanaka, 2007). In their model, the neurons of the granular layer receive sensory inputs, and generate long sequences of active neurons representing the passage of time. The activity of the granular layers neurons was read out at the olive, modeled by Purkinje cells. The functional role of the granular layers and Purkinje cells was regarded as a liquid state machine.

Rabinovich et al. (2008) showed that transient states can be useful to describe the neural network behavior. The idea of information processing with transient states was contrasted to processing with stable attractors. In the latter, the memory to a certain input is regarded as a stable state reached by the neural network after some transient behavior. However, the idea of reaching a certain attractor is sometimes not plausible in real and even artificial neural networks. Moreover, the experimental observations of the dynamics in the olfactory system of the locust support this alternative idea of computing with transient states (Rabinovich et al., 2008).

The ability of cortical areas to exhibit properties of a reservoir has been nicely demonstrated by Nikolić et al. (2009). In experiments where the responses of the primary visual cortex of anesthetized cats were recorded, it was found that a linear classifier is sufficient to properly classify the information about different stimuli. Furthermore, it was also found that new stimuli do not erase the information about previous ones, which can still be traced for several hundred milliseconds after they were switched off. They demonstrated that the neuron activity can be analyzed using linear classifiers and that the primary visual cortex exhibits fading memory properties, that are key in the reservoir computing paradigm.

More recently, Safaai and coworkers were exploring how texture is collectively encoded by populations of neurons in the barrel cortex of a rat (Safaai et al., 2013). They found that clusters of a few neurons can extract texture identity through a simple decoding scheme involving linear synaptic weighting, even in the case where no individual neurons represent the stimuli.

In summary, several experiments have been carried out during the last years, indicating how the brain acts as a complex, self-organized system in which parallel and sequential processing coexist in highly-interconnected networks. The observed dynamics, the high dimensionality of cortical networks, as well as the large amount of recurrent loops, might indeed suggest that the brain behaves similarly to a reservoir computer in the presence of sensory inputs (for review see Singer, 2013).

### 1.2. Hardware implementations employing delay-based reservoirs

In several applications, including handwriting recognition, time series prediction and others, RC has been numerically shown to outperform other computational approaches (Lukoševič et al., 2012). Finding suitable hardware for this scheme is crucial to better exploit the advantages of RC. Moreover, exploring the implications of information-processing in an analog manner, in particular in the presence of non-negligible noise, would advance our understanding of information-processing fundamentally. For this reason, hardware implementations are desired. However, traditional RC requires the realization of a network composed by a large number of interconnected neurons. Although the fixed connectivity structure of the recurrent network can be an advantage, a realistic hardware implementations remains challenging. In a pioneering work, Vandoorne et al. (2008) suggested RC as an appealing unconventional method to perform photonic information processing. The authors proposed the use of a network of semiconductor optical amplifiers. Although highly attractive, the technological requirements for this proposal are still very demanding. Instead, we present a radically different approach, based on delay-coupled dynamical systems, which simplifies the fundamental concept and its hardware implementations enormously without loosing performance.

**Figure 1:**
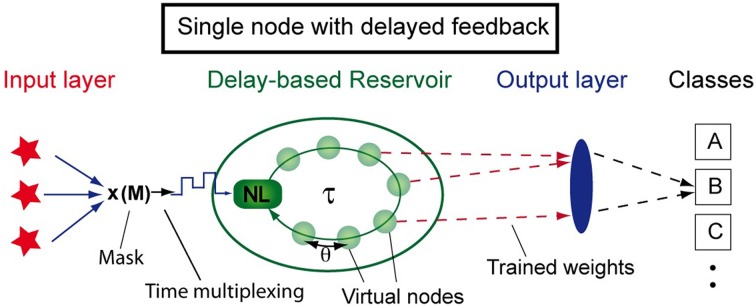
**Schematic arrangement of reservoir computing based on a single nonlinear node with delay and time-multiplexing**. Virtual nodes are defined as temporal positions along the delay line. Figure has been adapted from Appeltant et al. (2011).

In traditional reservoir computing, the network (or reservoir) is typically composed of spatially distributed nonlinear nodes with recurrent connections. However, other possibilities exist to emulate such a network. A powerful approach to achieve this is to replace the spatial distribution of nonlinear nodes by a single dynamical node with delayed self-feedback. Employing time-multiplexing, a network can be emulated, facilitating its hardware implementation as well as its fundamental understanding (Appeltant et al., 2011). Figure 1 depicts the general concept of this approach, including the three layers required for reservoir computing: the input layer, the delay-based reservoir and the output layer. In the delay-based reservoir, the recurrent connections are introduced by the self-feedback loop. Here, *virtual nodes* are defined as the states at equidistant temporal positions along the feedback loop. These virtual nodes, which are created via time-multiplexing, play a role analogous to the spatially distributed nodes in a traditional reservoir.

The delay-based implementation of reservoir computing fulfills the minimum requirements to perform information processing, namely the capability to map input states onto a high-dimensional state space, and to generate nonlinear transient responses. More specifically, delay systems are mathematically infinite-dimensional in the sense that any time-dependent solution is not uniquely determined by its initial state but depends on the continuous function of initial conditions in the range *t*=[−τ,0) (Erneux, 2009), where τ represents the delay time. Therefore, for large delay times, a single nonlinear dynamical node with delay can generate a sufficiently wide range of different transient responses (Dambre et al., 2012), which, at the same time, can be fully reproducible. The hardware implementation of delay-based reservoir computing is drastically simplified as compared to a realization of full networks. Consequently, the introduction of the delay-based reservoir computing concept has enabled a rapid advancement in the development of versatile hardware-based implementations of this machine-learning paradigm (Duport et al., 2012; Larger et al., 2012; Paquot et al., 2012; Brunner et al., 2013b).

## 2. Materials and methods

### 2.1. Delay-based reservoir computing

This minimal approach to information processing is based on the emulation of a recurrent network via a single nonlinear dynamical node subject to delayed feedback (Appeltant et al., 2011). As shown in Figure 1, we define *N* equidistant virtual nodes separated in time by θ = τ/*N* within one delay interval of length τ. The states of the *N* virtual nodes are defined as the values of the delayed variable at the corresponding temporal positions. These states characterize the transient response of the reservoir to a certain input at a given time. The temporal separation θ among virtual nodes cannot be chosen arbitrarily. It should neither be so long that the system reaches the steady state during this time period, nor should it be so short that the system has not been able to react to the perturbations. In fact, θ is a key parameter to optimize the reservoir performance. We typically choose 0.1 *T* < θ < *T*, with *T* being the characteristic time scale of the nonlinear dynamical node. Via this choice, the states of a virtual node depends on the states of previous neighboring nodes. Interconnected in this way, the virtual nodes emulate a network serving as reservoir (Appeltant et al., 2011; Schumacher et al., 2015).

**Figure 2:**
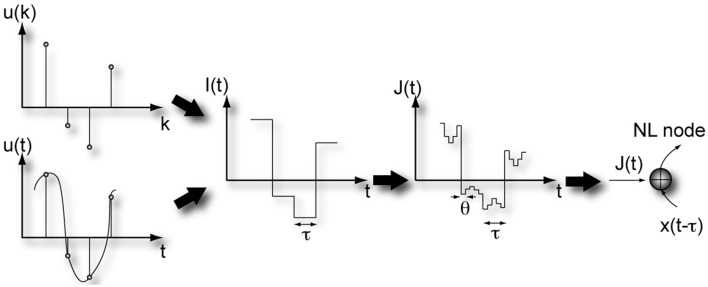
**Illustration of the masking steps required for information processing in a delay-based reservoir computer**. Figure has been adapted from Appeltant et al. (2011).

The reservoir, formed by the virtual nodes, is subjected to a time-continuous or time-discrete input stream, which can be a time-varying scalar variable or vector of any dimension. Individual virtual nodes are addressed by time-multiplexing the input signal. Then, to emulate the weights from the input layer to the reservoir in traditional RC, we introduce a mask. Figure 2 illustrates the masking procedure for a scalar input. First, the input stream undergoes a sample and hold operation to define a stream which is constant during one delay interval τ, before it is updated. Every segment of length τ is multiplied by the mask. Upon carrying out the multiplication of the mask with the sample at a certain time *t*_0_ of the input signal, we obtain a *N* dimensional vector which represents the temporal input sequence within the interval [*t*_0_, *t*_0_ + τ) (Appeltant et al., 2011). After a time τ, each virtual node in the delay line is updated.

More specifically, the dynamics of a reservoir composed by a nonlinear node subject to delay feedback can be described as (Appeltant et al., 2011; Schumacher et al., 2015)

(1) $$\dot{x}(t)=-x(t)+f(x(t-\tau ),J(t)).$$

In this equation *x* represents the dynamical variable of the nonlinear node, *x*(*t* − τ) is the delayed version of *x* at a certain past time τ, *f* is a smooth-real nonlinear function, and *J*(*t*) represents the input after pre-processing and time-multiplexing.

Once the input signal has been processed, a training algorithm assigns an output weight ω*_jk_* to each virtual node *x_j_* (*j* = 1…*N*), such that the weighted sum of the states approximates the desired target value *y_k_* as closely as possible,

(2) $$y_{k}=\sum_{j=1}^{N}\omega_{jk}x_{j}.$$

The training of the read-out weights follows the standard procedure for RC (Jaeger, 2001), e.g., linear regression. The testing is then performed using previously unseen input data of the same kind as those used for training.

### 2.2. Relevance of the input mask

The input mask serves three purposes: to sequentialize the input for the time-multiplexing, to maximize the (effectively used) dimensionality of the response, and to define the effective connectivity of the reservoir. In a traditional network approach, all the nodes in the reservoir can be addressed directly via direct connections from the input layer to the reservoir layer. In the delayed feedback approach, the input signal is fed into the nonlinear node, undergoes a nonlinear transformation, and then propagates along the delay line to the virtual nodes. The resulting connectivity resembles a ring-like topology with nearest and next-nearest neighbor coupling, which also in its explicit implementation has been shown to be very efficient for reservoir computing (Rodan and Tino, 2011).

By means of time multiplexing, the input signal, properly scaled, reaches the virtual nodes. Therefore, the input scaling needs to be imprinted before the signal is injected (see Figure 2). The input, after time multiplexing, consists of constant intervals θ corresponding to the separation between the virtual nodes in the delay line. Since different scaling factors are applied to the different virtual nodes, the reservoir state space is optimally explored.

When using a large number of virtual nodes, the mask can often be chosen as random. For a small set of nodes this choice can give rise to unsatisfactory results. Hence, a procedure to reliably assign mask values, such that a maximum diversity in reservoir states is created, is highly desired. Rodan and Tino (2011) showed that using aperiodic sequences in the input weights, deterministically generated from, e.g., a chaotic time series, could outperform random drawings. More targeted, Appeltant et al. (2014) outlined a procedure to reliably construct an optimal binary mask pattern optimizing the general reservoir response properties, derived from the concept of maximum length sequences. Although binary masks can have technical advantages in the context of delay-based reservoir computing, it is worth mentioning that multi-valued masks can improve the performance in some particular cases (Soriano et al., 2013) and continuous analog masks can simplify hardware implementations (Duport et al., 2014).

The precise timing between the input mask and the delay τ has a relevant impact on the ultimate performance of the reservoir. Two different strategies have turned out to be successful. Appeltant et al. (2011) proposed a mask length that matches the delay time, such that the connectivity between neighbouring virtual nodes is given by the inertia of the dynamical system. This requires the distance between the virtual nodes to be shorter than the characteristic time scale of the nonlinear dynamical node. In contrast, Paquot et al. (2012) proposed a mask length that is shorter than the delay time, such that the mismatch between mask length and delay time creates the connectivity between neighboring nodes. In consequence, the response of the nonlinear node can be considered as instantaneous. While the former method utilizes the given bandwidth of the system more efficiently, the latter allows for the use of a *static* nonlinearity.

**Figure 3:**
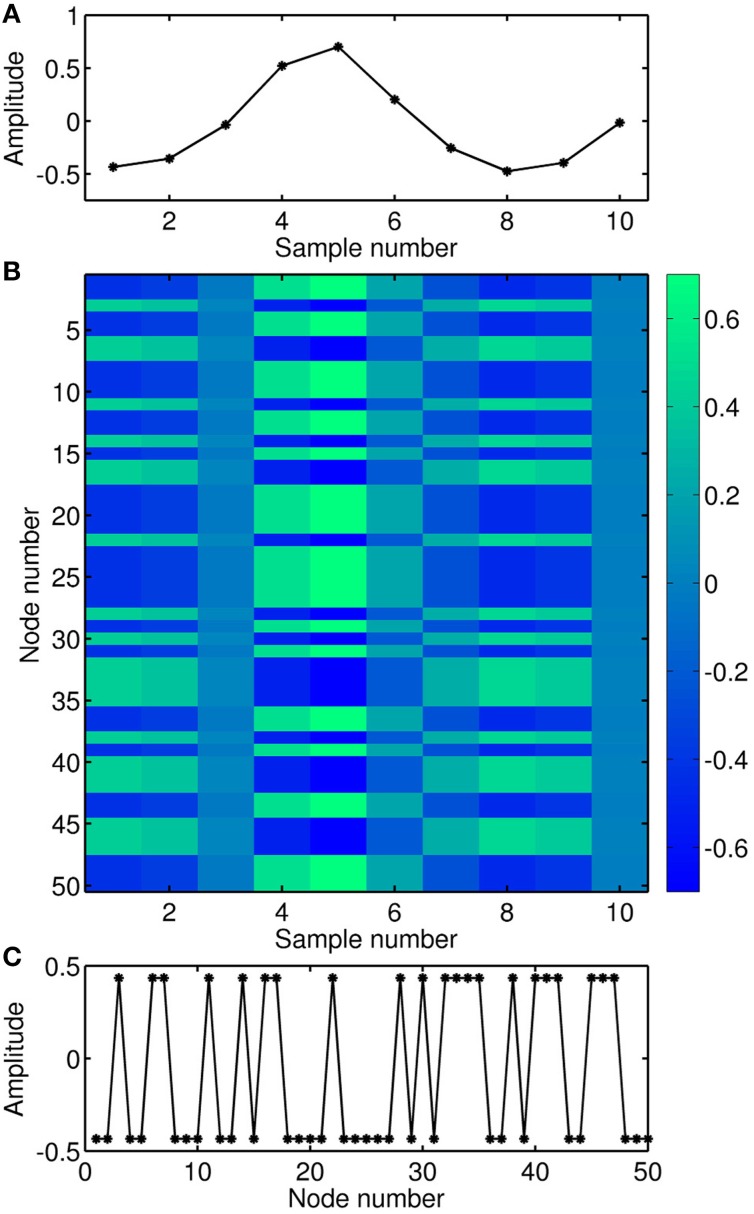
**Illustration of the input encoding**. **(A)** Temporal sequence of the input signal. **(B)** Matrix representation of the input signal multiplied by the mask, where the virtual nodes act as a pseudo-space. **(C)** Temporal sequence of the first sample of the input signal multiplied by the mask, i.e., expanded over the corresponding location of the virtual nodes.

Since the input in delay-based reservoir computing is time-multiplexed over the delay interval, the time within one delay interval can be interpreted as a pseudo-space. This interpretation is illustrated in Figure 3 for the case of a uni-dimensional input. In Figure 3A, 10 samples of a periodic input signal are depicted. Each sample is multiplied by an input mask and expanded over 50 virtual nodes. Figure 3B is a matrix representation of the input signal multiplied by the input mask. In this spatio-temporal matrix representation, the delay variable acts as a pseudo-space, and the color encodes the amplitude. For clarity, the first column in Figure 3B is shown in Figure 3C, where the mask (consisting of randomly alternating +1, −1) has been multiplied by the first sample of the input signal.

### 2.3. Nonlinear transient dynamics

Delay-based reservoir computing has also been referred to as nonlinear transient computing (Martinenghi et al., 2012; Brunner et al., 2013a). The term *nonlinear transient computing* puts emphasis on the origin of reservoir computing, namely the context-sensitive complex motion in a high-dimensional state space excited by the input signal. This interpretation has tight links to neuroscience. In particular, the olfactory network dynamics appears to classify different odors based on a coding space characterized by different complex transient spatio-temporal trajectories in the brain activity (Laurent, 2002). Key to this interpretation of reservoir computing is the fact that similar inputs create similar transient activity in the response of the system, a property referred to as reliability (Mainen and Sejnowski, 1995) or consistency (Uchida et al., 2004).

In order to illustrate the nonlinear transient dynamics in delay-based reservoir computing, we present a simple example, in which a *sin*^2^ function has been chosen as nonlinear function *f* in Equation (1). The dynamical evolution of the nonlinear node can then be described as follows,

(3) $$\dot{x}(t)=-x(t)+\beta sin^{2}(x(t-\tau )+J(t)+\phi ),$$

where β is the nonlinearity gain and ϕ is the offset phase.

**Figure 4:**
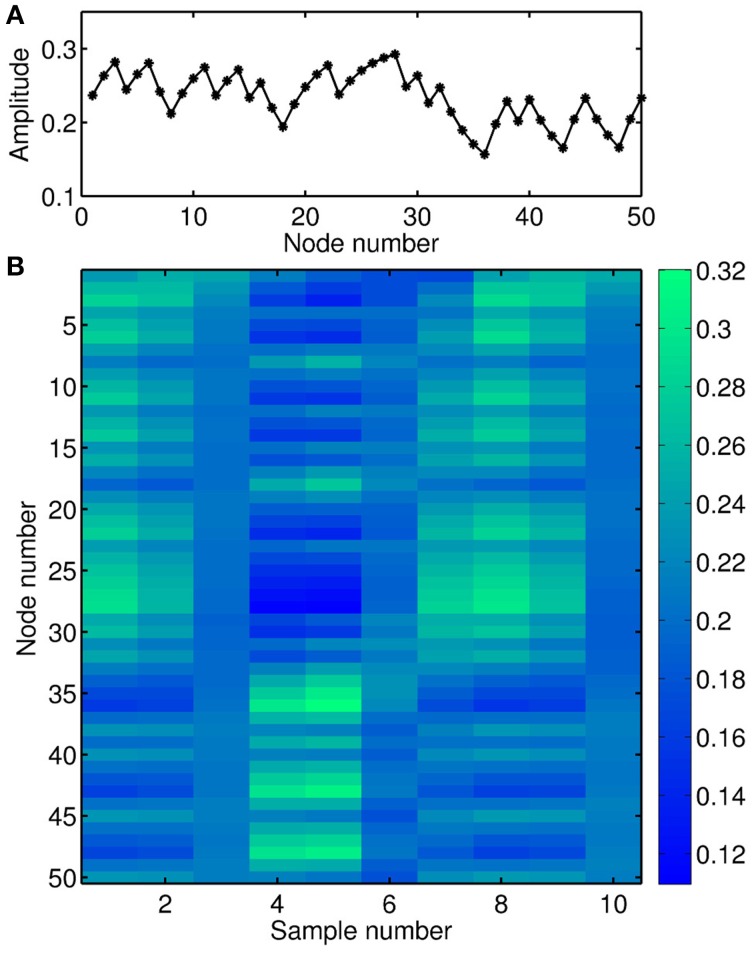
**Illustration of the nonlinear transient dynamics**. **(A)** Temporal sequence of the response to the signal depicted in Figure 3C. **(B)** Matrix representation of the response signal to the input matrix depicted in Figure 3B. Parameter values in Equation (3) are τ = 10, β = 0.7, and ϕ = -π/4, respectively.

Figure 4A shows the transient response of this nonlinear node to the input depicted in Figure 3C. In this example, the distance θ between the virtual nodes is 1/5 of the characteristic time scale *T* of the nonlinear node (*T* ≡ 1 in Equation 3, Larger et al., 2012). Thus, the nonlinear node is kept in a transient state and the value of a given virtual node depends on the values of previous neighboring nodes. Figure 4B is the spatio-temporal matrix representation of the response to the input depicted in Figure 3B. This response matrix, also known as state matrix *S_M_*, is later used for the classification of the input signal.

## 3. Results

### 3.1. Role of the nonlinearities

Reservoir computing based on a single dynamical node with delay has been realized in different experimental implementations, including electronic (Appeltant et al., 2011), opto-electronic (Larger et al., 2012; Paquot et al., 2012) and all-optical (Duport et al., 2012; Brunner et al., 2013b) hardware. Each of these realizations processes information based on hardware nodes with different types of nonlinearities. Electronic implementations have been based on a Mackey-Glass nonlinearity, the opto-electronics version on an Ikeda-type *sin*^2^ nonlinearity, while all-optical RC includes semiconductor lasers (Brunner et al., 2013b) and semiconductor optical amplifiers (Duport et al., 2012) as nonlinear nodes.

The number of the various nonlinearities for which successful RC has been implemented illustrates that the precise shape of the nonlinearity is not as crucial as originally assumed (Vandoorne et al., 2008). Nevertheless, depending on the specific task, performance does vary with the dynamical characteristics of the reservoir. Among the important properties of reservoir nodes is their balance between linear and nonlinear response. This dependence can be understood as follows: the more nonlinear a system, the easier it will be to linearly separate different input signals due to an increased dimensionality of its state space. In addition, nonlinearity simultaneously influences the autocorrelation properties of a delay system. Linear memory of delay systems manifests itself in delay echoes appearing in the autocorrelation function around multiples of the delay time τ. For a mostly linear system, these delay echoes can remain close to unity for *l* feedback delays with *l*>>1, illustrating the linear memory provided. The impact of nonlinearity in a delay-coupled system can be considered nontrivial (Porte et al., 2014a,b). However, in general terms, nonlinearity can cause the autocorrelation value to approach zero after only a small number of delays τ, and, therefore, typically limits the linear memory capacity (Jaeger, 2002). Computational performance thus depends on the particular requirements each task poses, highlighting the trade-off between memory and dimensionality expansion in delay systems with a single delay line.

Nonetheless, this trade-off does not represent a hard limit. Due to the simplicity of the delay systems, they can easily be extended by one or even multiple additional delay loops with different lengths. Experimentally, such a Reservoir Computer has been realized opto-electronically (Martinenghi et al., 2012). Following this approach, one can induce autocorrelation revivals at the delay times τ_*n*_ of the added feedback lines, significantly extending the linear memory capacity (Appeltant, 2012). Multiple delays therefore offer the possibility to tailor the contributions of nonlinearity and memory independently, allowing combinations which could not be achieved with the single delay system. This approach can even be employed for computational tasks which require memory for specific time scales.

In the opto-electronic system of Larger et al. (2012), the nonlinearity is based on the tunable interference between two optical waves within a Mach-Zehnder modulator, hence the opto-electronic nonlinearity resembles a sin^2^ function. Using a DC-bias, it is possible to modify the operating point (parameter ϕ in Equation 3), changing the conditions from strongly nonlinear around a local extrema of the sin^2^ function to predominantly linear at the position centered between a local minimum and a maximum. Therefore, by changing ϕ, one can evaluate the influence of linear and nonlinear response and their impact on computational performance for different tasks for otherwise identical systems.

**Figure 5:**
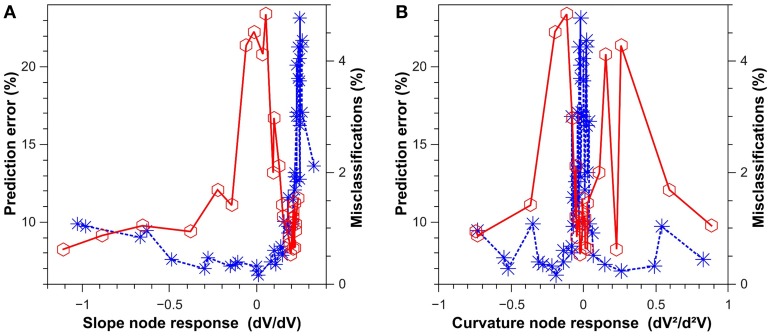
**Results for a classification task (blue asterisks) and a prediction task (red circles)**. **(A)** Performance of the system as a function of the slope of the node-response curve at the operating point. **(B)** Performance of the system as a function of the curvature of the node response at the operating point. The prediction task (SantaFe time series prediction) was evaluated via the normalized mean square error. The classification task (spoken digit recognition) was evaluated via the misclassification ratio. The results were obtained with the opto-electronic system introduced in Larger et al. (2012).

In Figure 5, we show the performance of a classification and a prediction task for a delay-based reservoir computer with an Ikeda-type sin^2^ nonlinear function *f* (see Equation 3). Blue asterisks represent the error rate for spoken digit recognition (classification), and red circles the standard deviation for the one-step prediction of a chaotic timeseries. For spoken digit classification, a subset of 500 isolated spoken digits were taken from the NIST TI-46 corpus database (Doddington and Schalk, 1981; Liberman et al., 1993). This set of the database contains ten spoken digits, from 0 to 9, which were recorded ten times each by five female speakers. To extract the best features for the classification, each sample was pre-processed using a cochlear ear model (Lyon, 1982). Here, 475 digits are used for training and 25 for the classification via cross validation. Using *y_k_* values from Equation (2), the reservoir selects the class *k* (*k* = 0…9) of the predicted digit using the winner-take-all (WTA) criterion. For the one-step prediction, the chaotic timeseries corresponds to the timeseries used in the Santa Fe timeseries prediction challenge (Weigend and Gershenfeld, 1991). The left panel depicts the task performance dependence on the linear response amplitude (local linear slope), and the right panel the task performances depending on the response curvature or nonlinearity around the operating point. In practice, the local slope and curvature of the nonlinear node is changed by controlling the phase offset ϕ of the sin^2^ nonlinearity. As can be seen for the classification data in Figure 5, the system performs well in classification tasks for a small linear response (slope node response), while a small nonlinearity (curvature node response) immediately leads to strong performance degradation. As it is the case for spoken digit classification, some classification tasks rely on a high-dimensional expansion of the injected information (strong nonlinearity), but they do not require a significant amount of memory (small linear response). Though the overall dependence is slightly more complicated, one finds that this dependency is inverted for the case of the chosen chaotic timeseries prediction task. Much more than the dimensionality expansion, chaotic timeseries prediction requires memory, and consequently prediction fails for a small linear response (slope node response) due to a reduction in reservoir memory.

### 3.2. Role of noise

In contrast to digital computing, the optical and electronic approaches described here compute in an analog manner, using the continuous values of the light intensity or voltage to encode data. This approach, thus, follows the ideas of alternative information processing paradigms (Crutchfield et al., 2010). In practical implementations of analog computing, the finite signal-to-noise ratio (SNR) may be a major limiting factor (Dambre et al., 2012). Several noise sources can be present at the different layers of the information processing system: noise can appear at the reservoir itself, and/or the input and output layers. In hardware systems, noise at the output layer is typically the strongest noise contribution to the signal (Soriano et al., 2013). The noise at the output layer has contributions from detection noise, measuring the response of the nonlinear node, and quantization noise, when converting the signal into a digital one.

Interestingly, classification tasks are relatively resilient against a finite SNR. For a spoken digit recognition task, an all-optical hardware system even outperformed software implementations of reservoir computing in terms of speed and accuracy (Brunner et al., 2013b). In contrast, the performance of other computational tasks, e.g., time-series prediction, significantly degrades when the SNR decreases (Soriano et al., 2013). In the latter case, the sensitivity of the performance to noise can still be minimized by using proper strategies.

Strategies to minimize the influence of noise can be implemented at the input pre-processing by optimizing the mask, or by exploring the optimum operating conditions for a given nonlinearity. In the case of mask optimization, input masks with multiple amplitude values can create reservoir responses that are easier to distinguish by the output layer, therefore minimizing the influence of detection noise (Soriano et al., 2013). In addition, some operating points are systematically more robust to noise for prediction tasks (Soriano et al., 2015). This enhanced robustness can be traced back to the properties of the nonlinearity around the operating point, highlighting again the relevant role of the nonlinearities. The combined use of these strategies is, to some extent, able to mitigate the performance degradation induced by noise. Such a performance degradation is not observed in numerical simulations with nearly infinite precision. This highlights the specific challenges when tackling information processing in analog systems in the presence of noise.

### 3.3. Understanding basic mechanisms

In order to solve a given computational task with reservoir computing, the parameters of the nonlinear node can be optimized recursively by trial and error or by using optimization algorithms. Since these optimization procedures are typically time-consuming, it is desirable to understand and quantify *a priori* information processing requirements. Therefore, several task-independent measures have been introduced to characterize computational ability and the memory of the system.

Regarding computational ability, the measures of kernel quality and generalization rank provide useful insights. For measuring the kernel quality, the reservoir state matrix *S_M_* is created for *N* different, random input vectors *U* = {*u*_1_, *u*_2_, *u*_3_, …, *u_N_*} with a length of *k* timesteps each. Using *k*−1 timesteps for warmup, the *k^th^* column of state matrix *S_M_* is concatenated for each of the *N* different input vectors, creating a matrix of dimension *N* × *N*. The kernel quality of the reservoir is then defined as the concatenated matrix rank *r_kq_*. In other words, the kernel quality characterizes how well the reservoir represents different input streams. As a computational scheme based on a linear separation via a hyperplane, RC ideally requires that the responses of the *N* different reservoir nodes are linearly independent (Legenstein and Maass, 2007). A well-suited reservoir is supposed to exhibit a rank of *r_kq_* → *N*, providing *N* linearly independent dimensions.

Next to the kernel quality, another crucial property is the ability to generalize (Legenstein and Maass, 2007). Ideally, the performance of a RC system should not be susceptible to very small differences, e.g., because of noise, as long as the general input pattern belongs to the same category. Again, state matrix *S_M_* is created for random input *U*' = {*u*'_1_, *u*'_2_, *u*'_3_, *u*'*_N_*}, each vector *u*'*_i_* of length *k*. Crucially, for this test, the final *l*-elements of each *u*'*_i_* are all replaced by the same random values *X* = {*x*_1_, *x*_2_, *x*_3_, …, *x_l_*}, such that the last *l* values of each *u*'*_i_* are identical. As before, we concatenate the *N* last columns of *S_M_*, creating a *N* × *N* matrix. The first *k*−*l* elements of the input data emulate the impact of noise, and, in a good reservoir, the last column of *S_M_* should be identical regardless of the previously injected noise-emulation. Thus, the generalization rank, again defined as the concatenated matrix rank *r_g_*, is supposed to be low for a reservoir with good generalization properties.

A good reservoir is simultaneously characterized by a high kernel quality rank and a low generalization rank, implying that if two inputs differ by more than a certain margin, they should be classified as different output classes. In turn, if the difference is inside this margin, they should be classified as the same class. When shifting the operating point of the nonlinear node toward regions where the separation to present inputs becomes larger, the separation of past inputs becomes larger as well, rendering the system less able to generalize. Therefore, it is desirable to work in parameter regimes that strike a balance between strong separation and sufficient generalization properties. To have an indication of the optimal regime for processing, the computational ability has been introduced as the difference between kernel quality rank and generalization rank (Legenstein and Maass, 2007; Appeltant, 2012). Thus, the computational ability of a reservoir is defined as

(4) $$r_{c}=r_{kq}-r_{g}.$$

Regarding the characterization of the memory of the system, the linear memory capacity quantifies the maximum memory of the system for random inputs. Using a random input sequence *u*(*k*), the reservoir's *i^th^* output *y_i_* is trained to reproduce the value of the information injected *i*-timesteps in the past (*u*(*k*−*i*)). The linear memory capacity μ*_c_* is then defined as the sum of the normalized, linear correlations between the shifted input information *u*(*k*−*i*) and the reservoir's trained classifier *y*_*i*_, letting *i* approach infinity (Jaeger, 2002; Verstraeten et al., 2007):

(5) $$\begin{array}{ll} m_{i} & =corr[y_{i}(k),u(k-i)],\\ \mu_{c} & =\sum_{1}^{\infty }m_{i}. \end{array}$$

When using a reservoir of size *N*, the maximum possible memory capacity equals *N*, a value that can be reached when using a purely linear reservoir (Jaeger, 2002).

For a good performance, a compromise between the memory capacity and the computational ability has to be found. As an illustration of this compromise, the region of good performance on the NARMA-10 task reported in Appeltant et al. (2011) corresponds to the region where both, memory and computational ability, score well (Appeltant, 2012).

**Figure 6:**
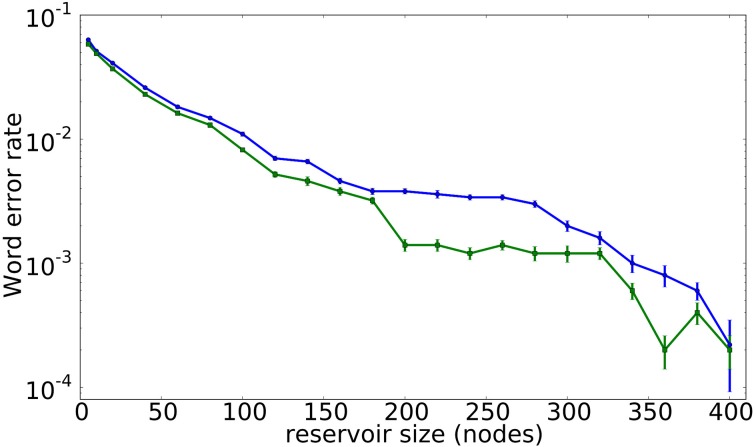
**Classification error rate for a spoken digit recognition task as a function of the reservoir size**. The blue line with dots corresponds to a reservoir of *N* nodes, while the green line with squares corresponds to a reservoir of 400 nodes in which only *N* nodes are available. The error bars are computed as the standard error over 10 realizations.

Naturally, the size of the reservoir has a strong influence on its computational performance. Figure 6 illustrates the evolution of the classification error for the spoken digit recognition task described in Section 3.1 as a function of the number of virtual nodes in the reservoir, with the classification error decreasing for an increasing *N*. More specifically, the blue line with circle symbols corresponds to a system with *N* virtual nodes, where all the node states are read out. In contrast, the green line with square symbols corresponds to a system with 400 virtual nodes, where only *N* nodes are accessible to the output layer. Interestingly, the results in Figure 6 suggest that a larger reservoir, even if only a part of the nodes are accessible for readout, performs better than a small reservoir. This finding resembles neuroscientific experiments in which brain signals can be reconstructed, even if only a reduced number of neurons are accessible, see e.g., Nikolić et al. (2009).

## 4. Discussion

### 4.1. Fundamental aspects

Understanding information processing in the brain is one of the most relevant challenges we face nowadays. The Human Brain Project, funded by the European Union, and the Brain initiative, funded by the USA, corroborate the importance and relevance of joining efforts to learn more about our brain. Remarkably, there is no broad consensus in the community to what extent recurrent loops play a significant role in brain circuits. Nevertheless, recurrent artificial neural networks have been designed and used to solve certain tasks that are inherently hard for traditional computers. Image and voice recognition, time series prediction and biomedical signal classification are only few examples of the successfully tackled tasks. Reservoir computing has emerged as one of the most promising and successful techniques for these tasks. Traditional reservoir computing uses the transient responses that inputs generate in a randomly-coupled neural network. This approach has certain similarities with some neuronal systems, e.g., in the olfactory system of the locust (Rabinovich et al., 2008) or the primary visual cortex of cats subject to visual stimuli (Nikolić et al., 2009). In reservoir computing, the transient responses, initially generated by some known inputs (learning procedure), are used to extract information from unknown input signals. Due to the complexity of the network and the input and output procedures it is difficult to gain insight into the basic mechanisms that allow such a powerful computation. In this paper we systematically reduced these ingredients to a simple system with the aim of uncovering the basic mechanisms that regulate its behavior. This minimal approach comprises a nonlinear node (a neuron) subject to a delay feedback loop. Despite its simplicity, this configuration offers remarkable results for certain tasks as pattern recognition or time series prediction. From a fundamental point of view, this approach opens perspectives to analyze and understand the essential ingredients and underlying mechanisms to such information processing concepts. Moreover, it allows to identify the role of important factors such as noise or heterogeneity, that are often neglected in digital concepts, but play an important role in the brain. The extension of the fundamental understanding, how computation can be efficiently and reliably performed in a heterogeneous and noisy environment, will be an important step toward decoding how information is processed in the brain.

Similar to traditional reservoirs, the delay-based approach is able to process information by means of its transient nonlinear responses. In addition, the delayed feedback creates a fading memory that allows to deal with context-dependent information. The spatio-temporal dynamical properties of delay systems allow for parallel and sequential information processing. A basic requirement to achieve competitive performances is to operate the system in a dynamical regime that generates reproducible and consistent transient responses.

Altogether, the minimal implementation of this neuro-inspired information processing approach offers an attractive solution for fundamental questions, as well as technological applications.

### 4.2. Applications and technological relevance

From a technological point of view, this minimal approach provides opportunities for implementations in various hardware platforms, either electronic, opto-electronic or all-optical, to perform computationally hard tasks. For all-optical implementations, channel equalization, radar, speech processing, or nonlinear time series forecasting are among the most attractive tasks (Duport et al., 2012; Brunner et al., 2013b). At the same time, simple operations such as vector and matrix multiplications are also feasible (Brunner et al., 2013a).

The software implementation counterpart of the single dynamical node with delay also offers interesting applications as a simple algorithm, for example to classify complex biomedical signals. This concept has been successfully applied to the detection of arrhythmias in electrocardiography signals (Lainscsek and Sejnowski, 2013; Escalona-Morán et al., 2014; Escalona-Morán et al., 2015).

### 4.3. Outlook

Employing a single dynamical node with delay is a simple, although powerful, solution to perform information processing and to understand the underlying mechanisms. In case the simple architecture of a single dynamical node with delay is not sufficiently powerful or fast enough to process complex signals, it can be combined with a hierarchical time-multiplexing structure (Zhang et al., 2014) or a combination of spatial- and time-multiplexing.

A promising extension to enhance the performance of this simple architecture is to introduce an additional feedback connection from the output layer back into the reservoir (Maass et al., 2007). This approach has been shown to increase significantly the fading memory of the system and improve its computational power. Moreover, connections between the output layer and the reservoir allow for online learning by e.g., optimizing an inverse model (Waegeman et al., 2012).

Delay-based dynamical systems are not only excellent platforms to implement the reservoir computing paradigm but they can also be treated as fully trainable recurrent neural networks using e.g., back-propagation methods (Hermans et al., 2015). This extends the range of practical problems in which the minimal approach can achieve state of the art performance. In addition, an unsupervised reservoir adaptation through various forms of synaptic plasticity, such as spike-time-dependent plasticity, can also be implemented (Paugam-Moisy et al., 2008). Adaptation to the task at hand is again a neuro-inspired solution that can clearly improve the performance of the system.

Finally, the Boolean world is also a candidate to implement reservoir computing techniques (Snyder et al., 2013). In this case, the Boolean logic is not used to implement standard sequential programming but to define a random network of elements that behave in an autonomous manner. Such applications are already being developed in the sense of traditional reservoir computing (Snyder et al., 2013) and delay-based reservoir computing Haynes et al. (2015), respectively. These techniques appear as an extension to the conventional use of Boolean systems, and their possibilities are largely unexplored.

Altogether, we are convinced that the cross-fertilization between neuroscience, machine learning and dynamical systems offers a promising path, not only to build better information-processing systems, but potentially to learn more about how our brains perform many tasks in such a successful manner.

## Author contributions

All authors contributed to the discussion of the results and to the writing of the manuscript.

### Conflict of interest statement

The authors declare that the research was conducted in the absence of any commercial or financial relationships that could be construed as a potential conflict of interest.
